# Supplementary material for: Successional Shifts in Soil C:N:P stoichiometry and its environmental controls during forest restoration in Huize County, Qujing, Yunnan Province
Source: PLoS One. 2026 Aug 21;21(8):e0356261. doi: 10.1371/journal.pone.0356261 (PMC13496410; doi:10.1371/journal.pone.0356261)
Supplement: Supplementary file 2 [file pone.0356261.s004.zip › Data and Code/RF/Pinus yunnanensis forest/figures/PDP_C.P_s.TP.pdf]

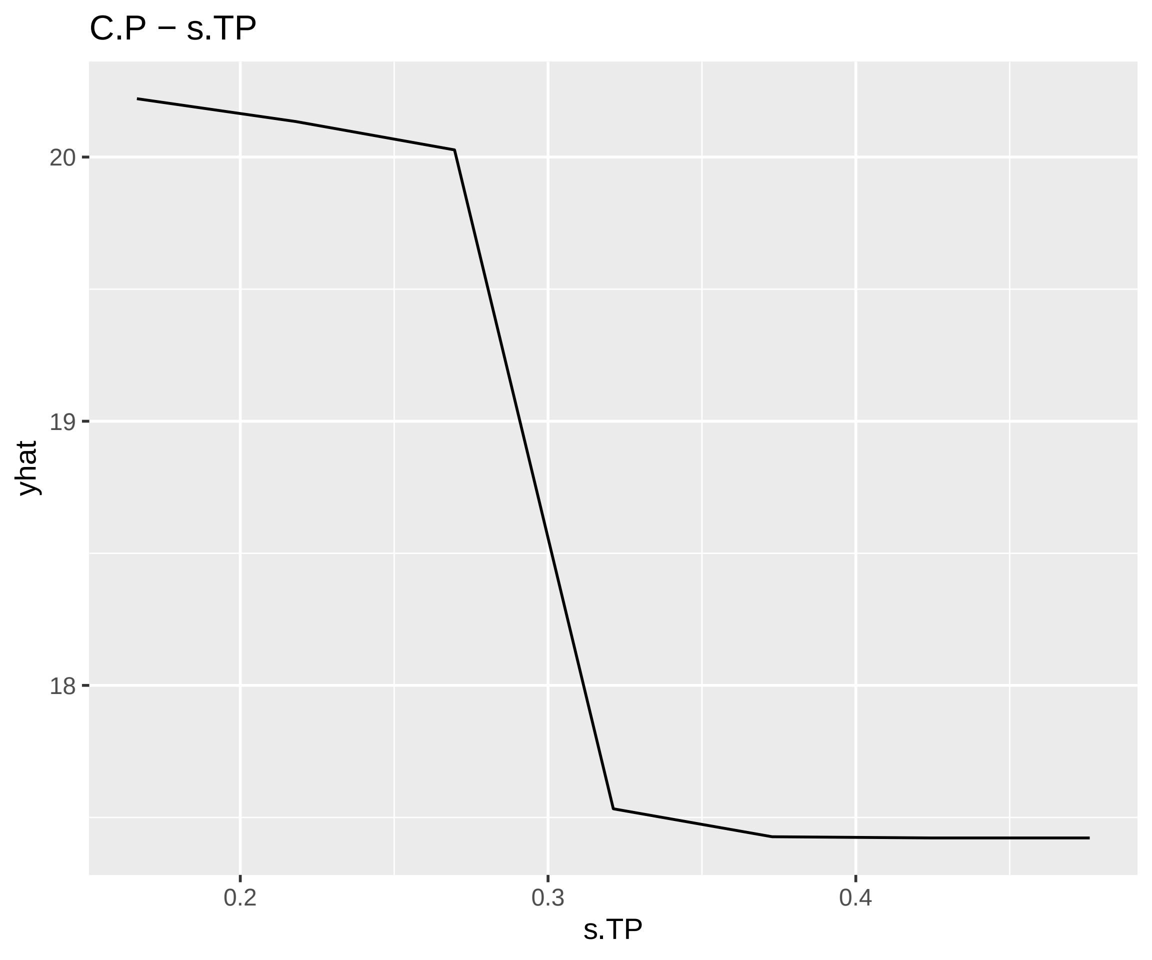
C.P − s.TP
20
19
18
yhat
0.2
0.3
0.4
s.TP
